# The effects of gravity and compression on interstitial fluid transport in the lower limb

**DOI:** 10.1038/s41598-022-09028-9

**Published:** 2022-03-22

**Authors:** James W. Baish, Timothy P. Padera, Lance L. Munn

**Affiliations:** 1 Department of Biomedical Engineering, Bucknell University, Lewisburg, PA USA; 2 Department of Radiation Oncology, Massachusetts General Hospital and Harvard Medical School, Boston, MA USA

**Keywords:** Biomedical engineering, Mechanical engineering, Computational models

## Abstract

Edema in the limbs can arise from pathologies such as elevated capillary pressures due to failure of venous valves, elevated capillary permeability from local inflammation, and insufficient fluid clearance by the lymphatic system. The most common treatments include elevation of the limb, compression wraps and manual lymphatic drainage therapy. To better understand these clinical situations, we have developed a comprehensive model of the solid and fluid mechanics of a lower limb that includes the effects of gravity. The local fluid balance in the interstitial space includes a source from the capillaries, a sink due to lymphatic clearance, and movement through the interstitial space due to both gravity and gradients in interstitial fluid pressure (IFP). From dimensional analysis and numerical solutions of the governing equations we have identified several parameter groups that determine the essential length and time scales involved. We find that gravity can have dramatic effects on the fluid balance in the limb with the possibility that a positive feedback loop can develop that facilitates chronic edema. This process involves localized tissue swelling which increases the hydraulic conductivity, thus allowing the movement of interstitial fluid vertically throughout the limb due to gravity and causing further swelling. The presence of a compression wrap can interrupt this feedback loop. We find that only by modeling the complex interplay between the solid and fluid mechanics can we adequately investigate edema development and treatment in a gravity dependent limb.

## Introduction

As relatively tall mammals, humans have developed adaptations to manage the effects of gravity on the fluid balance in our tissues which, if unopposed, can add about 75 mmHg of pressure for each meter of height. Normally, interstitial fluid pressure (IFP), even in the human lower leg, is held at or just below atmospheric pressure by a tightly regulated balance between losses from the blood vessels and clearance by the lymphatic vessels^1–3^. Unfortunately, there are several pathologies that can disrupt the normal fluid balance leading to fluid accumulation—edema—which if left unchecked can lead to recurrent skin and soft tissue infections, chronic wounds and loss of the limb function. Chronic edema or lymphedema is reported to impact about 2 to 4% of the population^4^ and 24 to 49% of radical mastectomy patients^5^. Lymphedema accounted for an estimated 165,055 hospital admissions in the United States between 2012 and 2017 ^6^. Despite the apparent, widespread prevalence of this debilitating disorder, firm statistics that could support better public health attention continue to be studied^7^.

The rate at which interstitial fluid arises from the blood vessels can be increased by either increases in the pressure in the capillaries or increases in the permeability of the capillary walls. Normally, the capillary wall forms a relatively tight barrier to fluid loss, and the capillary bed is shielded from high pressure in the arteries by precapillary sphincters and by one-way valves in the veins that prevent the pooling of blood from above. But during angioedema or inflammation the capillary wall becomes more permeable to plasma, leading to an increased source of interstitial fluid even when the capillary pressure is normal. Alternatively, trans-wall flow from the capillaries can be increased by higher capillary pressure when the venous valves fail to prevent back flow—a condition known as venous insufficiency^8–10^—which affects more than 36% in the general population over age 40 with increasing prevalence with age^11^.

Up to their limits, lymphatic vessels can respond to an increased source of interstitial fluid with stronger pumping. However, too little lymphatic clearance—called lymphedema may occur from surgical removal of lymph nodes, parasitic obstruction, congenital defects or other damage to lymphatic vessels^12,13^. In the early stages of lymphedema, fluid accumulates in the subcutaneous tissues. As the lymphedema progresses into later stages, the fluid inundated tissue is replaced with adipose tissue^14^.

Gravity imposes local challenges to maintaining tissue-fluid balance through its contributions to higher capillary pressure and the added difficulty of pumping lymph fluid uphill. In addition, gravity has a more global effect by directly pulling fluid downward through the interstitium. Here, we will consider both local and global effects of gravity on tissue-fluid balance.

When edema does arise, mechanical compression serves as one of the few clinical tools available to manage the swelling in the limbs^15–26^. While known to be effective, mechanisms by which compression manages the fluid balance in the leg are only partially understood.

Previous computational analyses have addressed many aspects of fluid management in the leg, but none have been sufficiently comprehensive to allow a full discussion of the competing processes and the use of compression in edema. Typically, the tissue is modeled as a saturated poroelastic material that is divided into solid and fluid subvolumes. At the local scale (about 1 cm) poroelastic methods have been employed in the contexts of solid tumors^27–33^, injection sites^34–39^, under indentation probes^40–42^, near the edge of a compression wraps and in normal tissues^43–47^. The local models often include capillary permeability and lymphatic clearance, but over such relatively short distances, gravity has little opportunity to build significant hydrostatic pressures. Large-scale models of the entire limb in compression may include gravity, tissue elasticity, and sometimes even include large-scale percolation, but typically neglect local capillary and lymphatic source/sink effects^48–56^.

Another key feature of the fluid balance in the leg is that the tissue permeability (or equivalently the hydraulic conductivity) depends strongly on the state of strain in the tissue^57–60^. Guyton^61^ found that the volume of an excised dog leg increased rapidly when the IFP slightly exceeded atmospheric pressure. He also found that the easy dilation of the leg in this range of pressures was accompanied by an increase in tissue permeability of as much as five orders of magnitude due to expansion of available pore spaces in the interstitium^57^. More generally, the size of tissue pore spaces is determined by a balance between the IFP in the tissue pores which tends to dilate the tissue and compression of the solid subspace by external loading from gravity or wraps.

Our present goal is to incorporate all of the aforementioned features, albeit each in simplified form, to better understand the relative contributions of each effect on lower leg edema.

## Model formulation

**Figure 1:**
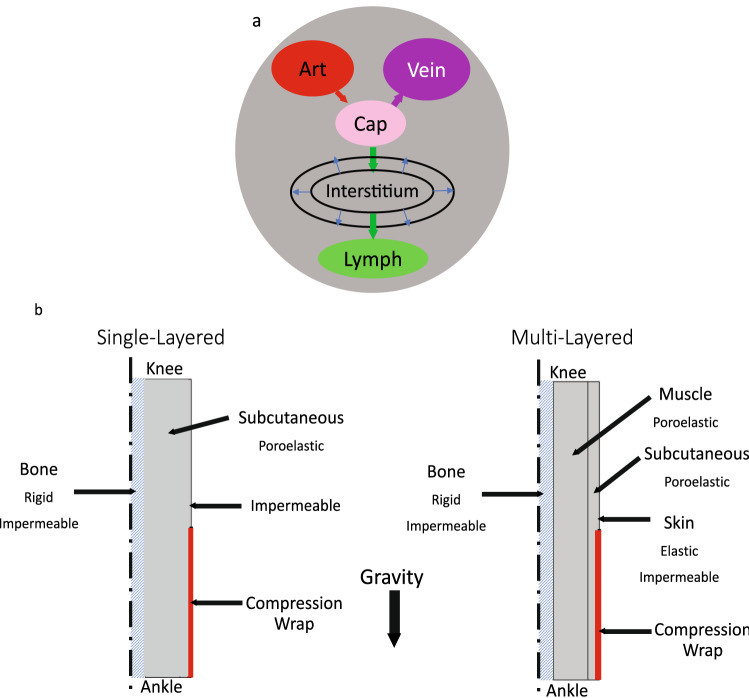
Conceptual model and geometry. (**a**) The local fluid exchange between the interstitium and the capillaries and initial lymphatic vessels is represented as a distributed source/sink without explicitly representing individual blood or lymph vessels. (**b**) Simplified, axisymmetric geometries of the lower leg used in the model. The bone is assumed to provide a rigid, impermeable surface at the inner boundary of the soft tissues. The soft tissue may move radially and vertically at the upper and outermost boundaries, but can move only radially at the lower boundary. Fluid can enter or leave the system locally via the blood and lymph vessels or through the interstitium at the upper boundary. All other boundaries are impermeable.

To that end, we represented the lower leg as a cylindrical bi-phasic porous medium (Fig. 1). The idealized geometry allows the physical processes to produce relative changes in the dimensions during swelling and treatment (Fig. 1). The governing equations that follow can be readily adapted to more anatomically detailed models if desired. A single-layered model allowed the consideration of a homogenous material, while the multilayered model introduced effects arising from tissue-dependent properties in the skin, subcutaneous tissue, and muscle. Each tissue was modeled as a continuum consisting of a fluid phase and a solid phase. Both blood and lymphatic vascular effects were accounted for as distributed sources or sinks of fluid in the continuum model where fluid percolates throughout the interstitium or contributes to a local expansion of the tissue volume. While the vessels were not accounted for explicitly, the effects of solid stress on their pressures and pumping capabilities can be incorporated. The IFP from the fluid phase acts on the solid phase as a pore pressure that tends to expand the tissue.

## Governing equations

### Fluid phase

The fluid velocity $\mathop{u}\limits^{\rightharpoonup}$ is assumed to be driven by gradients in the IFP *p*_*i*_ and by gravity based on Darcy’s law^62^

1 $$ \mathop{u}\limits^{\rightharpoonup} = - \frac{\kappa }{\mu }(\nabla p_{i} + \rho \mathop{g}\limits^{\rightharpoonup} ) $$

where $\kappa$ is the interstitial permeability of the tissue, $\mu$ is the fluid viscosity, and $\mathop{g}\limits^{\rightharpoonup}$ is the downward-acting acceleration of gravity. The pressure *p*_*i*_ in Eq. (1) is a scalar quantity that represents only the pressure due to the fluid phase as distinct from the compressive stress that can be present in the solid phase. The ratio $\kappa /\mu$ is frequently called the hydraulic conductivity.

Assuming the same density in the fluid and solid phases, mass is conserved by

2 $$ \nabla \cdot \mathop{u}\limits^{\rightharpoonup} + \frac{\partial \phi }{{\partial t}} = J_{c} + J_{L} $$

where $\phi$ is the tissue porosity which can change as fluid is forced into or out of the pore space and where *J*_*c*_ and *J*_*L*_ are the distributed capillary and lymphatic sources or sinks of fluid, respectively (source when positive, sink when negative). Starling’s law is used to represent the capillary source^63^

3 $$ J_{c} = \beta_{c} (p_{c} - p{}_{i}) $$

where $\beta_{c} = \left( {L_{p} S_{v} } \right)_{c}$ is the product of the capillary permeability and the surface area per unit volume. Under normal conditions, the capillary pressure is tightly regulated by precapillary sphincters in the arterioles and by one-way valves in the veins that shield the capillaries from hydrostatic pressures arising from uninterrupted columns of fluid in the veins above. When the valves in the veins malfunction during venous insufficiency we can include a gravity-induced gradient ($g_{c} > 0$) in the vertical (*z*) direction $p_{c} = p{}_{c0} + \rho g_{c} (h - z)$. The reference capillary pressure *p*_*c*0_ can include osmotic effects $p_{c0} = p_{0} - \sigma \Delta \pi$ where *p*_0_ is the hydrostatic pressure, $\sigma$ is the osmotic reflection coefficient and $\Delta \pi$ is the osmotic pressure difference) and is assigned at the upper boundary. A similar, simplified model of lymphatic pumping is used

4 $$ J_{L} = \beta_{L} (p_{L} - p_{i} ) $$

where $\beta_{L}$ is an effective lymphatic conductance with lymphatic pumping normally able to create a net suction effect ($p_{L} < 0$), ^64^ which if reduced has been demonstrated to contribute to edema^65^. While more sophisticated models of lymphatic pumping are available, this two-parameter ($\beta_{L} ,p_{L}$) model allows us to explore loss of lymphatic function by reducing $\beta_{L}$ or by making *p*_*L*_ less negative to yield less suction or even positive to apply a retrograde pressure. Analogous to the capillary pressure, the lymphatic pressure can include gravity effects $p_{L} = p{}_{L0} + \rho g_{L} (h - z)$. Equilibrium at atmospheric pressure $p_{i} = 0$ requires that $J_{c} + J_{L} = 0$, implying $\beta_{c} p_{c} = - \beta_{L} p_{L}$ under normal conditions.

### Solid phase

The total stress in the solid phase $$\overset{\lower0.5em\hbox{$\smash{\scriptscriptstyle\rightharpoonup}$}}{\overset{\lower0.01em\hbox{$\smash{\scriptscriptstyle\rightharpoonup}$}} {\sigma}}_{total}$$ is taken to be the sum of the fluid phase stress tensor $$\overset{\lower0.5em\hbox{$\smash{\scriptscriptstyle\rightharpoonup}$}}{\overset{\lower0.01em\hbox{$\smash{\scriptscriptstyle\rightharpoonup}$}} {\sigma}}_{fluid} = - p_{i} \overset{\lower0.5em\hbox{$\smash{\scriptscriptstyle\rightharpoonup}$}}{\overset{\lower0.01em\hbox{$\smash{\scriptscriptstyle\rightharpoonup}$}}{I}}$$ from the pore pressure and the solid phase stress tensor $$\overset{\lower0.5em\hbox{$\smash{\scriptscriptstyle\rightharpoonup}$}}{\overset{\lower0.01em\hbox{$\smash{\scriptscriptstyle\rightharpoonup}$}} {\sigma}}_{solid}$$. Under the influence of gravity, the force balance is written as

5 $$ \nabla \cdot \overset{\lower0.5em\hbox{$\smash{\scriptscriptstyle\rightharpoonup}$}}{\overset{\lower0.01em\hbox{$\smash{\scriptscriptstyle\rightharpoonup}$}} {\sigma}}_{total} + \rho \mathop{g}\limits^{\rightharpoonup} = \nabla \cdot (\overset{\lower0.5em\hbox{$\smash{\scriptscriptstyle\rightharpoonup}$}}{\overset{\lower0.01em\hbox{$\smash{\scriptscriptstyle\rightharpoonup}$}} {\sigma}}_{solid} - \alpha p_{i} \overset{\lower0.5em\hbox{$\smash{\scriptscriptstyle\rightharpoonup}$}}{\overset{\lower0.01em\hbox{$\smash{\scriptscriptstyle\rightharpoonup}$}}{I}} ) + \rho \mathop{g}\limits^{\rightharpoonup} = 0 $$

where we take the Biot-Willis coefficient $\alpha$ to be unity. Assuming a homogeneous, isotropic material the stress and strain are related by

6 $$ \overset{\lower0.5em\hbox{$\smash{\scriptscriptstyle\rightharpoonup}$}}{\overset{\lower0.01em\hbox{$\smash{\scriptscriptstyle\rightharpoonup}$}} {\sigma}}_{solid} = 2G\overset{\lower0.5em\hbox{$\smash{\scriptscriptstyle\rightharpoonup}$}}{\overset{\lower0.01em\hbox{$\smash{\scriptscriptstyle\rightharpoonup}$}} {\varepsilon}} + (K - \frac{2G}{3})tr(\overset{\lower0.5em\hbox{$\smash{\scriptscriptstyle\rightharpoonup}$}}{\overset{\lower0.01em\hbox{$\smash{\scriptscriptstyle\rightharpoonup}$}} {\varepsilon}} )\overset{\lower0.5em\hbox{$\smash{\scriptscriptstyle\rightharpoonup}$}}{\overset{\lower0.01em\hbox{$\smash{\scriptscriptstyle\rightharpoonup}$}}{I}} $$

where *G* is the shear modulus and *K* the bulk modulus under the condition of zero pore pressure. The parameters *G* and *K* are convenient here due to our emphasis on the change in volume as the tissue is dilated or compressed by fluid and/or stress effects. They may be related to the familiar Young’s modulus *E* and Poisson’s ratio $\upsilon$ by

$$ G = \frac{E}{2(1 + \upsilon )}\quad {\text{and}}\quad K = \frac{E}{3(1 - 2\upsilon )} $$

We take the dilatory strain $$e = tr(\overset{\lower0.5em\hbox{$\smash{\scriptscriptstyle\rightharpoonup}$}}{\overset{\lower0.01em\hbox{$\smash{\scriptscriptstyle\rightharpoonup}$}} {\varepsilon}} )$$ to be isotropic so that

7 $$ e = tr(\overset{\lower0.5em\hbox{$\smash{\scriptscriptstyle\rightharpoonup}$}}{\overset{\lower0.01em\hbox{$\smash{\scriptscriptstyle\rightharpoonup}$}} {\varepsilon}} ) \approx \frac{\Delta V}{{V_{0} }} $$

The tissue dilation may occur in a somewhat anisotropic manner, but including directional effects is beyond the scope of the available measurements. Likewise, the analytical results presented here are based on a linear elastic material that facilitates dimensional analysis, but the numerical implementation readily permits hyper-elastic constitutive formulations.

**Figure 2:**
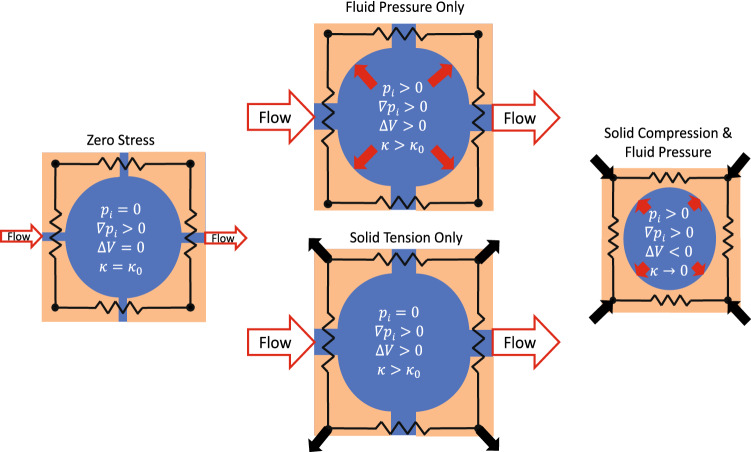
Schematic of tissue permeability dependence on volume changes that arise from the balance of fluid pressure and solid-state stress. A zero solid-state stress condition on the left is assumed to occur when IFP is near atmospheric pressure in the absence of external loading. Changes in either IFP or solid stress can change the pore space through which fluid can percolate leading to higher flow rates with the same gradient in IFP. Under conditions of sufficient solid-state compression on the right, the flow paths are highly restricted leading to minimal flow and the potential for increased fluid pressure.

When the tissue dilates either from elevated interstitial pressure or volumetric stretching of the solid subvolume, the pore space available for fluid movement *e* increases leading to increased interstitial permeability^57^ (Fig. 2). Following methods adopted in other tissues, we model this effect with^35,60,66–71^

8 $$ \kappa = \kappa_{0} \exp (Me) $$

where $\kappa_{0}$ is the tissue permeability under baseline conditions and the parameter *M* determines the strength of the strain effect on $\kappa$.

### Characteristic length and time scales

Dimensional analysis of the governing equations with constant coefficients yields characteristic length and time scales that facilitate our examination of the most dominant effects in a given case. Two characteristic length scales arise from the governing equations. The first, which we will call the vascular absorption length, is the typical distance over which a pressure difference between the vasculature (blood and lymphatic) and the interstitium can persist before equilibrium is reestablished. Here we adopt

9 $$ L_{v} = \sqrt {\frac{\kappa }{{\mu (\beta_{c} + \beta_{L} )}}} $$

akin to the formulation previously employed in the literature^28,29,43,72^. Now that we include the effects of gravity, we introduce an additional length scale relating the tissue stiffness to the specific gravity of the tissue

10 $$ L_{g} = \frac{K}{\rho g} $$

which we will call the gravity length. The gravity length is a measure of how much the interstitial pore size is increased by hydrostatic pressure. Comparing these two lengths yields a dimensionless ratio $L_{v} /L_{g}$ that determines whether interstitial fluid enters or exits the vasculature freely enough to avoid undergoing a substantial increase in hydrostatic pressure. That is, small $L_{v} /L_{g}$ indicates that the IFP is driven mostly by the local balance between the capillaries and the lymphatic vessels. Whereas, large $L_{v} /L_{g}$ suggests that interstitial fluid is not exchanged fast enough to avoid large scale, vertical percolation of interstitial fluid along the limb.

There are also two relevant characteristic time scales^28,29^. The vascular absorption time scale corresponding to *L*_*v*_ is

11 $$ \tau_{v} = \frac{1}{{K(\beta_{c} + \beta_{L} )}} $$

and the interstitial percolation time scale is

12 $$ \tau_{i} = \frac{{\mu L^{2} }}{\kappa K} $$

where *L* is the distance over which fluid must transport. These parameters were first applied to solid tumors^27–33^ and injection sites^34–39^ where *L* was on the order of 1 cm. Whereas, here the distance that would be needed to force fluid from beneath a compression wrap would be considerably larger, perhaps approaching the height of the wrap on the limb—i.e., tens of centimeters. When we introduce estimated parameter values, we see that movement of fluid solely via percolation along a limb through the interstitium is much slower than vascular interactions unless local absorption by the vasculature is impeded.

### Numerical implementation

The model was implemented in COMSOL®5.5 using the CFD and structural mechanics modules. Poroelasticity is modeled with quasistatic, linear elastic solid mechanics that is linked to fluid modeled by a fully transient Darcy’s law by using the IFP as the pore pressure in the solid. Gravity is applied in the solid and fluid subvolumes. IFP dependent mass sources are added to Darcy’s law to model the capillary and lymphatic interactions. The compression wrap is modeled as an inward acting pressure on the lower portion the leg. Other boundary conditions are as described in Fig. 1. An extremely fine, physics-controlled triangular mesh is used. A mesh size study assured good convergence.

### Parameter values

The dimensions and estimated parameter values for a baseline case are available in Table 1. In general, the baseline values serve to demonstrate how the length and time scales we have introduced govern the key processes involved. Then as we will show, the effects of the wide physiological range and uncertainty in some of these parameters can be readily explored by hand calculations of the relevant scales without need to reproduce a full numerical simulation. Estimates of the bulk modulus, tissue permeability, vascular permeabilities and lymphatic suction pressure are the most important for our purposes.

**Table 1:** Baseline parameter values.

Parameter	Definition	Value	Units	Tissue
*h*	Height	0.5	m	
*r*_*bone*_	Bone radius	0.01	m	
*t*_*muscle*_	Muscle thickness	0.05	m	
*t*_*subcut*_	Subcutaneous tissue thickness	0.02	m	
*t*_*skin*_	Skin thickness	0.002	m	
*ρ*	Density	1000	kg/m^3^	All
*μ*	Viscosity	0.001	Pa-s	All
*g*	Acceleration of gravity	9.81	m/s^2^	All
*G*	Shear modulus	3.45 × 10^4^	Pa	Skin
		8.00 × 10^2^		Subcutaneous
		8.62 × 10^3^		Muscle
*K*	Bulk modulus	1.00 × 10^6^	Pa	Skin
		1.33 × 10^3^		Subcutaneous
		8.33 × 10^4^		Muscle
*κ*_0_	Interstitial permeability at zero strain	0	m^2^	Skin
		1 × 10^−14^		Subcutaneous
		1 × 10^−14^		Muscle
*M*	Interstitial permeability strain coefficient	12	1	Subcutaneous
				Muscle
*L*_*p*_	Capillary permeability	0	m/Pa-s	Skin
		2.71 × 10^−11^		Subcutaneous
		2.71 × 10^−11^ ^27^		Muscle
*S*_*v*_	Capillary surface area per unit volume	0	m^−1^	Skin
		7,000		Subcutaneous
		7,000 ^27^		Muscle
*β*_*L*_	Lymphatic conductance	0	1/Pa-s	Skin
		9.47 × 10^−8^		Subcutaneous
		9.47 × 10^−8^ ^27^		Muscle
*ϕ*	Porosity^1^	0.05	1	Subcutaneous
		0.05		Muscle
*p*_*c*0_	Reference capillary pressure	1333 (10)	Pa (mmHg)	All
*p*_*L*0_	Reference lymph pressure	−266 (−2)	Pa (mmHg)	All
*p*_*wrap*_	Wrap pressure	1333 (10)	Pa (mmHg)	

^1^The porosity provided here is a nominal initial value. The final results are insensitive to this value since the tissue permeability is calculated dynamically as a function of volumetric strain with Eq. (8).

The bulk modulus of the tissues can vary widely depending on the loading conditions. Guyton^61^ found that for IFP just above atmospheric pressure the volume of the dog hindlimb increased by about 10% for each mmHg increase in IFP under acute conditions ($K \approx 1330\;{\text{Pa}}$). An even lower modulus (higher apparent compliance) has been reported in the human arm with chronic edema^73^ ($K \approx 350\;{\text{Pa}}$). There are other reports^74,75^ of tissue compliance so high that the bulk modulus is nearly zero. Bates^73^ points out that most of the volume change in the limb occurs in the subcutaneous adipose tissue, making the compliance of the subcutaneous layer even higher than that for the limb as a whole. For subatmospheric pressures, Guyton found that the tissues became much stiffer when most of the free fluid was removed. Other studies of adipose tissue in compression also find much higher stiffnesses in compression than in the early stages of extension^76–81^. At large tensile strains exceeding 30% the adipose tissue again stiffens as fibrous elements in the tissue come into tension^81^. We assume that most of the change in volume due to fluid accumulation occurs in the subcutaneous layer which is largely adipose tissue. In the single-layer model, we use the properties of the subcutaneous layer throughout. In the multi-layer model, muscle^82,83^ and skin^84–90^ have stiffer characteristics than the subcutaneous layer.

Guyton^57^ also found that the interstitial permeability of tissue was a strong function of IFP when no external constraint such as a wrap was present. He reported increases in $\kappa$ of as much as five orders of magnitude as the IFP increased above atmospheric. Here we use $\kappa = \kappa_{0} \exp (Me)$ as suggested by Mow and Lai^68^. Setting the parameter $M \approx 12$ yields a variation consistent with the observed bulk modulus. As we will show, the results will depend strongly on the estimated values for $\kappa_{0}$, *K*, and *M*, all of which can vary widely and none of which are known with high certainty.

## Results

With the aid of the length and time scales introduced above, we wish to address two broad questions. First, under what conditions does gravity matter? And second, with the addition of compression where does the fluid go and how long does it take to get there?

We begin with a healthy, baseline condition in which a local balance between capillary leakage and lymphatic clearance $J_{c} = - J_{L}$ exists in the absence of large-scale percolation. Here we expect from Eqs. 3 and 4 that IFP is determined by

13 $$ p_{i} = \left( {\beta_{c} p_{c} + \beta_{L} p_{L} } \right)/\left( {\beta_{c} + \beta_{L} } \right) $$

**Figure 3:**
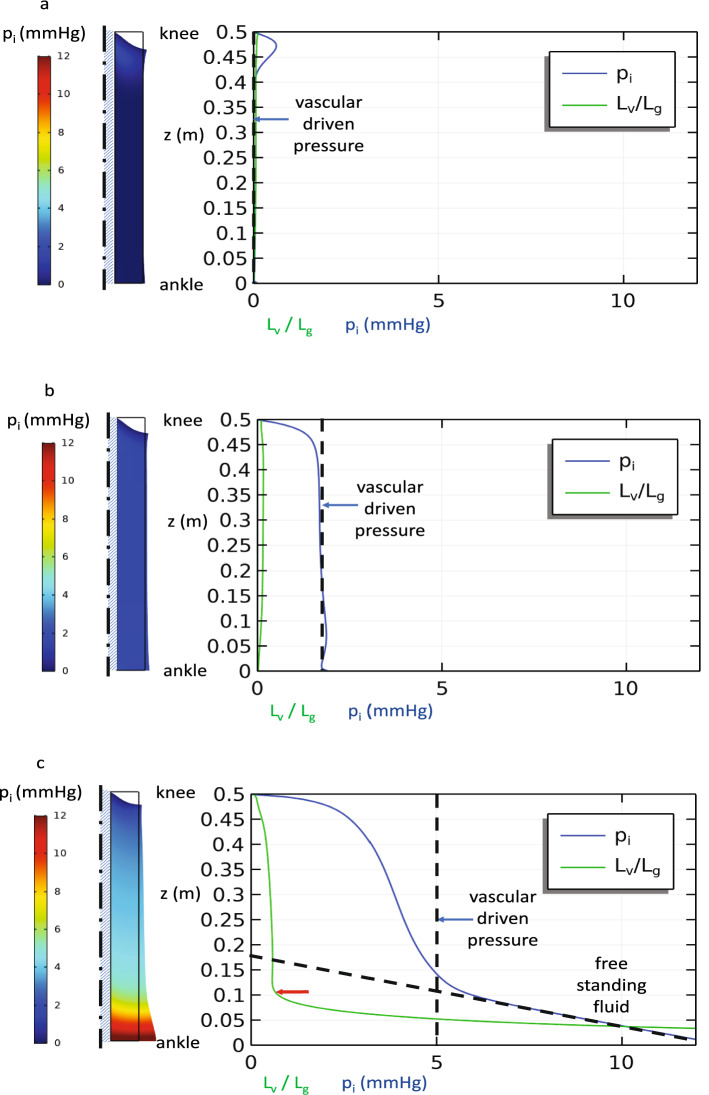
Baseline steady state simulation of the single-layer model with constant lymphatic clearance parameters, but increasing capillary pressure: (**a**) p_c_ = 10 mmHg (**b**) p_c_ = 20 mmHg, and (**c**) p_c_ = 40 mmHg. IFP and $L_{v} /L_{g}$ plotted along the outer surface of the leg. The dashed lines show the IFP expected due to a strictly local balance between the interstitial space and the vasculature as predicted by Eq. (12) at 0 mmHg for p_c_ = 10 mmHg, 2 mmHg for p_c_ = 20 mmHg, and 5 mmHg for p_c_ = 20 mmHg, and a free-standing column of fluid in (**c**). A transition from local vascular-driven pressure to limb-scale, free-standing fluid is apparent when $L_{v} /L_{g}$ increases significantly due to increased interstitial permeability when the tissue expands from high internal pressure below z = 0.1 m at the red arrow in (**c**).

IFP can be held near atmospheric pressure $p_{i} = 0$ by $\beta_{c} p_{c} = - \beta_{L} p_{L}$ provided that the pumping capacity of the lymphatic vessels is sufficient to keep up with the fluid leaking from the local capillaries. Under these conditions, we observe (Fig. 3a) some sagging of the soft tissue under its own weight as the central bone supports most of the soft tissue at each level. The tissue is stretched somewhat artificially near the upper boundary by its weight due to the free condition applied there, whereas tissue near the ankle is compressed by the weight of the tissue above. However, neither fluid induced-swelling nor large-scale percolation of fluid occur since $L_{v} < < L{}_{g}$ (Table 2). In Fig. 3b and c we see that increases in capillary pressure without commensurate increases in lymphatic clearance capability leads to uniform increases in IFP and modest changes in tissue volume until the IFP is large enough to induce significant increases in tissue permeability $\kappa$. A steady, local fluid balance may still be maintained near the knee if the tissue dilation does not increase $\kappa$ too greatly, but Fig. 3c shows that as the IFP increases near the ankle, so does the vascular absorption length *L*_*v*_. When the fluid must percolate relatively far before vascular absorption can take place, gravity has enough vertical distance to build hydrostatic pressure $L_{v} /L_{g} = O(1)$. We see that the IFP in Fig. 3c no longer tracks that suggested by Eq. (13) in the lower leg. Instead, below this transition, the IFP increases as it would in a free-standing pool of fluid with the vascular interactions becoming secondary. The region near the ankle would then act like a water-filled balloon. (We note that the transition does not occur at precisely $L_{v} /L_{g} = 1$, but closer to $L_{v} /L_{g} \approx 1/2$ due to a small multiplicative factor not revealed by dimensional considerations alone.)

**Table 2:** Baseline values of length and time scales.

Parameter	Symbol	Value
Vascular absorption length	$L_{v}$	0.0094 m
Gravity length	$L_{g}$	0.14 m
Vascular absorption time	$\tau_{v}$	6.6 × 10^3^ s
Interstitial Percolation time	$\tau_{i}$	4.7 × 10^6^ s

**Figure 4:**
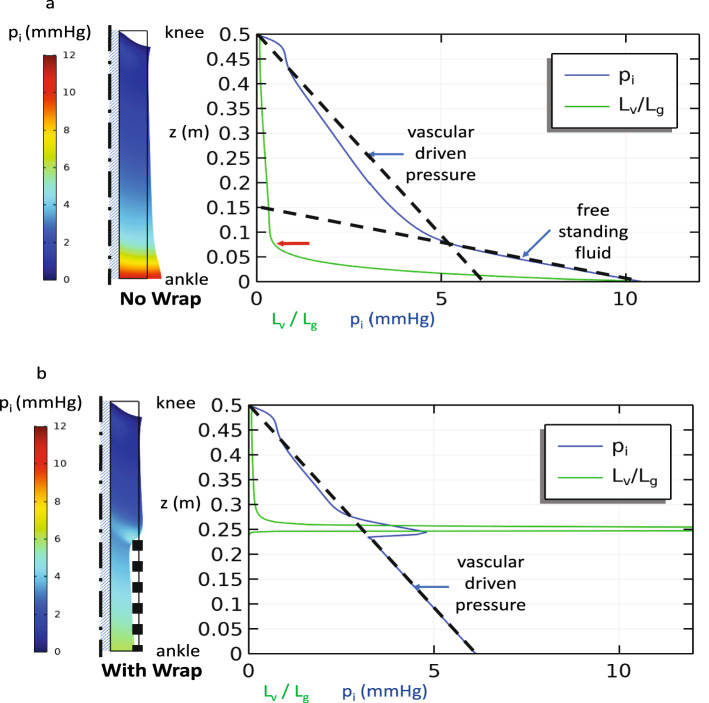
Steady state simulation with venous insufficiency in a single-layer model: p_c_ = 10 mmHg + $\rho$ g(h-z) and p_L_ = −2 mmHg. Interstitial pressure along the outer surface and $L_{v} /L_{g}$ (**a**) without and (**b**) with a compression wrap that imposes a radial pressure, but does not alter the capillary pressure by relieving venous insufficiency. The dashed lines show the pressure gradients expected due to a free-standing column of fluid and that due to a strictly local balance between the interstitial space and the vasculature. A transition from local vascular driven pressure to limb-scale free standing fluid is apparent when $L_{v} /L_{g}$ increases significantly due to increased interstitial permeability when the tissue expands from high internal pressure below the red arrow (**a**) or imposed local solid stress at the upper edge of the wrap (**b**).

Conditions of lymphedema may be modeled by any combination of factors that limits the ability of the lymph vessels to match the supply of interstitial fluid from the capillaries. For example, while Fig. 3 shows the effects of increased capillary pressure without compensatory increases in lymphatic clearance, nearly identical pressures arise when the capillary pressure is held constant, but the lymphatic suction pressure is reduced (increased *p*_*L*_) or the lymphatic clearance rate constant $\beta_{L}$ is reduced. Likewise, angioedema or inflammation that can increase the permeability of the capillary wall $\beta_{c}$ will lead to excess swelling only if the lymphatic clearance cannot compensate. Here again the results follow exactly the same trend as those in Fig. 4 as $\beta_{c}$ is increased.

Another pathology of interest is venous insufficiency (Fig. 4a) where failure of the valves that prevent back flow of blood in the veins allows an uninterrupted column of fluid to develop in the veins. Here the capillary pressure will increase with distance below the knee according to $p_{c} = p{}_{c0} + \rho g_{c} (h - z)$. If the lymphatic clearance as represented by $\beta_{L} p_{L}$ can adjust to the increased load, then the IFP can be maintained at atmospheric pressure. However, if the lymphatic clearance parameters reach a constant, limiting value, then the IFP will increase along with $p_{c}$ according to Eq. (13) as it did in Figs. 3b and c. Figure 4 shows that as IFP builds and the tissue dilates, a positive feedback process can occur as increased $\kappa$ leads to $L_{v} /L_{g} = O(1)$. Beyond this threshold, the interstitial fluid again pools in the lower leg.

The addition of a compression wrap on the lower leg (Fig. 4b) can compress or prevent the expansion of the interstitial pore space, which in turn lowers $\kappa$ thus offsetting the effect of gravity in venous insufficiency. The only exception is in a small region near the upper edge of the wrap where the tissue bulges outward giving a locally increased tissue permeability that is resolved under the wrap. If in addition, compression restores venous valve function—effectively eliminating the gravity effects in the capillaries ($g_{c} = 0$)—we return to the baseline conditions in Fig. 3a where $p_{i} \approx 0$. In our example, we have applied a relatively mild compression pressure (10 mmHg) that is sufficient to reverse the dilation of the interstitial pore spaces present in highly compliant, fluid-saturated subcutaneous tissues. As most of the interstitial fluid is displaced by the compression, we would expect the tissue to substantially stiffen against further compression requiring much higher compression pressures to induce further volume reduction. Clinically, pressures in the range of 30 mmHg or more are commonly used for compression garments^23,91^.

**Figure 5:**
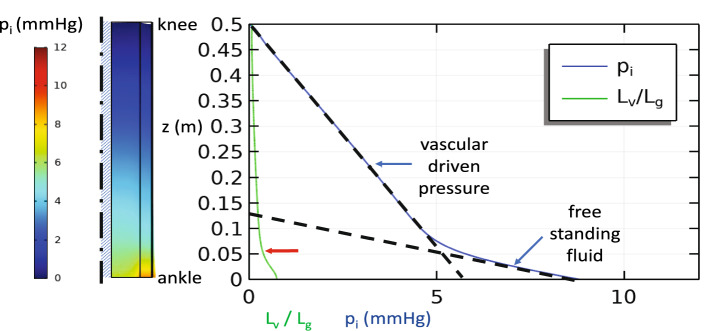
Multi-layer model with venous insufficiency. Parameter values plotted in subcutaneous layer. Note how the transition to free-standing fluid occurs lower on the leg (red arrow) than in Fig. 4a.

In Fig. 5, we show the effects of partitioning the soft tissue into skin, subcutaneous, and muscle layers where the subcutaneous layer is the region of greatest compliance and fluid mobility. Adding the skin layer increases the overall stiffness of the soft tissue—effectively increasing *L*_*g*_ which lowers where on the leg the free fluid percolation through the interstitium begins.

**Figure 6:**
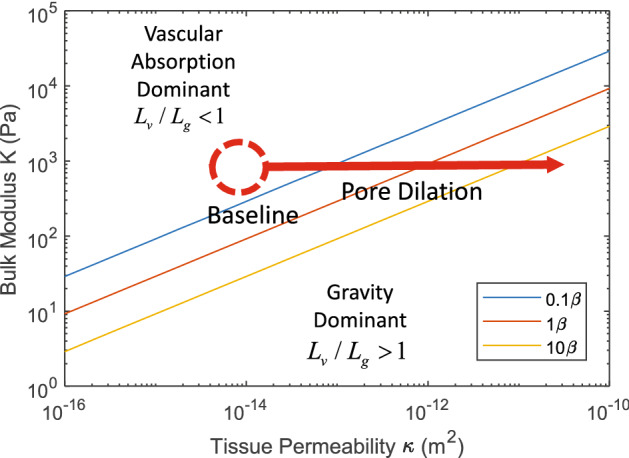
Map based on Eq. (14) showing how vascular absorption dominates at the baseline conditions of high tissue stiffness *K* and low tissue permeability $\kappa$, while gravity-driven pooling in the interstitium dominates for low tissue stiffness and high tissue permeability that can arise from dilation of the interstitial pore space. The lines of constant $\beta = \beta_{c} + \beta_{L}$ determine when transition from vascular absorption dominance to gravity dominance occurs with the baseline conditions indicated by $1\beta$.

Having shown that the ratio $L_{v} /L_{g}$ determines the dominant processes of fluid movement in the limb, an exhaustive parametric study by numerical methods is unnecessary. Instead we can examine how the ratio

14 $$ \frac{{L_{v} }}{{L_{g} }} = \frac{\rho g}{K}\sqrt {\frac{\kappa }{{\mu (\beta_{c} + \beta_{L} )}}} $$

depends on each of the relevant parameters. Since density, gravitational acceleration, and the viscosity are effectively constant only the tissue permeability, tissue compliance and the capillary and lymphatic exchange rates matter. Figure 6 shows the combination of parameter values that leads to either local vascular absorption dominance (upper left) or limb-scale, gravity-driven pooling (lower right). The greatest risk of gravity-driven pooling occurs when the tissue is highly compliant (low stiffness) and is highly permeable—a circumstance that is most likely when unconstrained adipose tissue is exposed to IFP’s just above atmospheric pressure. Transition to gravity-driven pooling occurs with lower tissue permeability or stiffer tissues when the vascular clearance parameter $\beta = \beta_{c} + \beta_{L}$ is reduced. Any intervention that stiffens the tissue, compresses the tissue, decreases capillary pressure or increases lymphatic clearance has the promise of offsetting the tendency of fluid to accumulate low in the leg. These findings are consistent with known interventions for edema, helping give confidence in our model.

**Figure 7:**
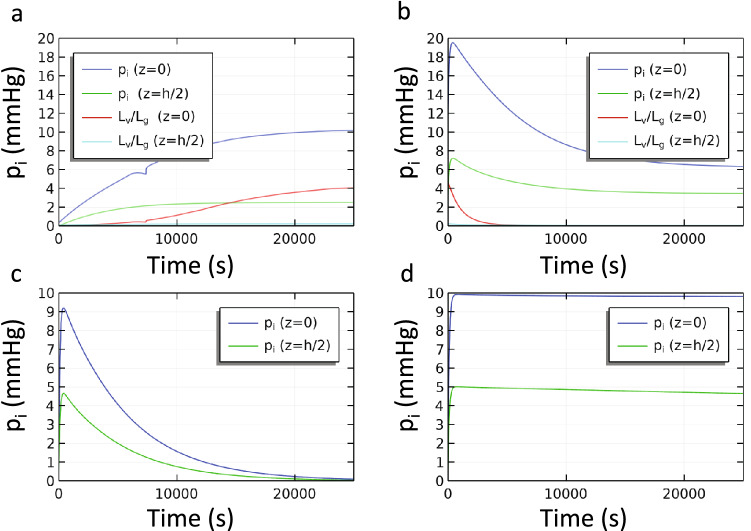
Transient single-layer model with baseline parameters. The IFP is shown at the ankle level (z = 0) and at the top of the wrap (z = h/2). (**a**) No wrap is yet present. The initial condition is the steady solution with constant capillary pressure from Fig. 3a (p_c_ = 10 mmHg). At t = 0 venous insufficiency begins. The final condition is that in Fig. 4a. (**b**) A compression wrap (0 < z < h/2) is applied gradually during the first 100 s to the final condition from (**a**). The final condition in (**b**) is shown in Fig. 4b. (**c**) Single-layer model without gravity with baseline vascular interactions and (**d**) without gravity and without vascular interactions ($\beta_{c} = \beta_{L} = 0$). Values for $L_{v} /L_{g}$ remain low throughout for panels **c** and **d**.

Next, we consider the time course of the development of edema and its response to compression. First, we determine how much time is needed for dilation of the ankle area to occur when a hydrostatic gradient is initially added to the capillary pressure in the unwrapped leg as would occur in venous insufficiency (Fig. 7a). Effectively, how long can a patient with venous insufficiency remain vertical before swelling ensues? We find that the IFP increases on a time scale dominated by vascular absorption since $L_{v} /L_{g}$ is initially small through the leg. The baseline value of the vascular absorption time ($\tau_{v}$ from Eq. (11)) constant is about 7,000 s which is consistent with the result in Fig. 7a. Interstitial percolation is initially very slow, but accelerates later in the transient as the local values of the tissue permeability and $L_{v} /L_{g}$ increase near the ankle. Coincidently in this case, the deviation from smooth increases in pressure occurs around 7,000 s when the local value of $L_{v} /L_{g}$ approaches unity for the first time. When a compression wrap is applied to the lower half of the leg in Fig. 7b the IFP undergoes an initial spike after which the IFP relaxes gradually to the final conditions in Fig. 4b on a time scale that is again dominated by vascular absorption ($\tau_{v}$).

Removing the effects of gravity, but retaining the wrap, allows a direct comparison between vascular absorption and interstitial percolation rates. Figure 7c and d show how IFP at the ankle and at the top of the wrap returns to normal after a wrap is applied. Under these conditions, given enough time IFP will return to atmospheric pressure throughout the leg. Fluid must either be locally absorbed or squeezed to above the wrap through the interstitium. In Fig. 7c the time scales estimated from Eqs. (11) and (12) using the baseline parameter values are $\tau_{v} \approx 7 \times 10^{3} \;{\text{s}}$ and $\tau_{i} \approx 10^{7} \;{\text{s}}$ where we assume that the distance *L* over which percolation must occur is on the order of 0.25 m. When vascular absorption is active, as it is in Fig. 7c, the transient occurs at the time scale dominated by local absorption. In contrast, elimination of the vascular absorption pathway in Fig. 7d greatly slows the relaxation of pressures to the time scale of fluid percolation from beneath the wrap to the upper leg. We also note that despite the strong dependence of both time scales on the tissue stiffness, their relative values are unchanged since they share the same dependence on *K.* Only when local lymphatic clearance is significantly reduced does interstitial percolation occur fast enough to dominate.

**Figure 8:**
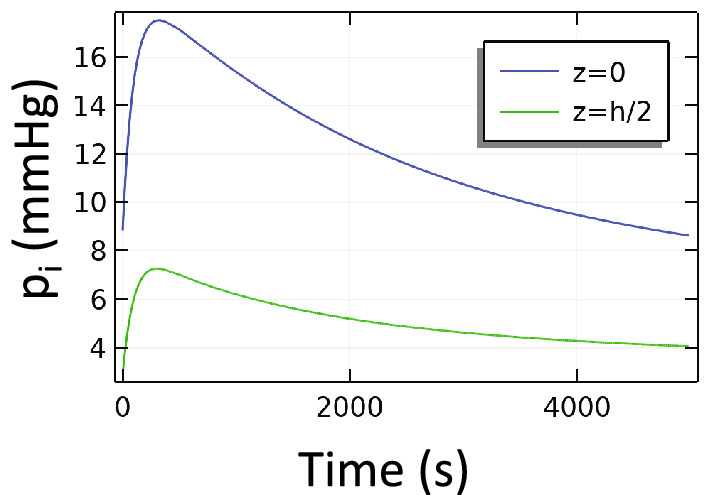
Transient multi-layer model with compression. Beginning with an initial condition in which venous insufficiency produced swelling near the ankle (z = 0), a compression wrap (0 < z < h/2) was applied gradually over the first 100 s. At the last time point shown, the IFP is still relaxing to its steady state value. The time scale here is dominated by the vascular absorption process.

In Fig. 8 we consider the multi-layer model. Here we see that IFP responds somewhat faster than the single-layer model which lacks skin. As we observed in the steady state in Fig. 5, the addition of skin increases the overall stiffness of the tissue. The effect of increased stiffness on the transient response is to decrease both vascular absorption and interstitial percolation times—speeding the approach to equilibrium.

## Discussion

Management of lower limb edema is largely empirical in the clinic, with insufficient theoretical foundation to guide therapy. Here, we have presented a mathematical model that includes the key mechanical considerations and fluid dynamics that are responsible for maintaining tissue fluid homeostasis under normal physiological conditions—and that are disrupted in lower limb edema. The model shows that IFP is normally kept at a low level by a local balance between the blood and lymphatic vasculatures: fluid lost from the blood is quickly absorbed by nearby lymphatic vessels. Only under extreme conditions of tissue dilation do the tissue permeability and tissue compliance reach levels that allow interstitial fluid to move freely enough for hydrostatic effects to become significant within the interstitium itself. However, even when interstitial fluid movement is relatively local, gravity effects can initiate edema by increasing the pressure—and leakage from—the blood capillaries and/or by reducing lymphatic clearance.

Comparisons with experimental reports in the literature support our findings. Christensen et al.^92^ found that patients with primary lymphedema in one leg had subcutaneous IFP of 14.8 mmHg when supine and 17.9 mmHg when standing, while the IFP in the healthy leg remained just below atmospheric and did not change significantly with position. Similarly, they found that post-thrombotic syndrome (which impairs venous function) led to IFP in the affected leg of 2.8 mmHg when supine and 4.0 mmHg when standing with IFP remaining just below atmospheric in the healthy leg in both positions. Bates et al.^73^ also found that lymphedema in the arm following breast cancer surgery elevated the IFP by 3.5 mmHg above normal. Chronic kidney disease also produces edema and increased IFP in the leg to 5.5 mmHg above normal^93^. Our numerical predictions are consistent with these results—introduction of vascular and lymphatic pathologies raises the IFP and the imposition of gravity further exacerbates these effects.

One of the most common strategies for controlling edema is so-called RICE (rest, ice, compression and elevation). Our results directly address the effects of a compression wrap and the gravitational effects of elevation. The imposed external compression can have multiple effects. Probably the most important effect is to improve the function of the one-way valves in the veins, which has the beneficial effect of decreasing backflow into the capillaries and the consequent increase in fluid pressure and leakage. In the present study, we did not model the mechanics of the veins and their valves explicitly, but rather assigned the capillary pressure according to whether the limb had functional valves (capillary pressure was constant) or whether there was venous insufficiency (a hydrostatic gradient developed along the leg). Another important effect of compression is that it directly deforms and confines the interstitium. Immediately after application of compression the IFP may undergo a temporary spike before the fluid is either absorbed by the local vasculature or has time to percolate to sites not under the compression (Fig. 8). In the longer term, the dimensions of the pores available for fluid percolation are reduced, leading to significantly lower tissue permeability, which restricts fluid to only local interactions with the vasculature. Once locally constrained, the interstitial fluid cannot generate a standing pool of liquid with gravity-induced gradients.

Compression wraps are generally uncomfortable, and patient compliance is a challenge, so optimizing their effectiveness would be beneficial. Our analysis revealed important dynamics occurring at different time scales that can help guide this optimization. Key considerations are the time course of compression treatment and the eventual disposition of the fluid. For example, knowing how long the fluid takes to move in response to the compression wrap (hours or days, or even weeks) would help determine whether it would be better to apply a stiff wrap that loosens as the leg dimensions diminish (and must be reapplied frequently) or to use a more adaptable/flexible/elastic wrap that holds a more nearly fixed pressure with respect to leg dimension. Optimization of intermittent/peristaltic and graded compression technologies will also benefit from a better understanding of the time course of fluid movement in the limb.

Another component of RICE is icing, or cooling. The poroelastic perspective utilized here yields some insight into the effect of tissue cooling, even though temperature was not explicitly modeled. Cooler weather has been associated with less swelling in lymphedema patients^94^. In addition, locally cooled tissues became softer as measured by indentation even though there was little change in fluid volume^95^. Such reduced resistance to indentation might be explained by a favorable combination of changes in the interstitial fluid pressure, the interstitial fluid mobility and the solid tissue stiffness. Interstitial fluid pressure can be reduced by lowering the capillary blood pressure via thermoregulatory vasoconstriction or decreasing the permeability of the capillary walls as it might be with reduced inflammation^96^. Another possible softening mechanism that is little explored is a reduction in fibroblast tensioning within the tissue which may alter the pressure–volume relationship^97^. Offsetting these favorable effects are the possible deductions in lymphatic pumping^98^, increases in fluid viscosity and higher stiffness of the solid phase^99^, all of which tend to increase, rather decrease, the apparent stiffness of the tissue when cooled. Given that lower apparent stiffness is associated with positive clinical outcomes, further attention to the role of managing the interstitial fluid pressure and tissue stiffness with treatment would seem to be warranted. The present poroelastic model promises to be a useful tool for examining the tradeoffs among the various competing effects.

Because it includes gravitational effects, it is interesting to use our model to analyze how animals of different sizes achieve tissue fluid homeostasis. Gravitational effects should be minimal in mice, where the largest vertical distance is on the order of a few centimeters (this must be carefully considered when extrapolating conclusions regarding edema and tissue drainage obtained using murine models). Giraffes, on the other hand, are tall enough to generate hydrostatic pressures over several meters. Consequently, they have multiple adaptations to manage vascular and interstitial pressures^100–108^. Specifically, the lower leg of the giraffe has thick arterial walls, tight capillary walls, and relatively stiff skin and connective tissues all of which protect the giraffe from excess pooling of fluid in the leg. IFP in the giraffe leg is normally elevated (44 mmHg^100^) well above that in healthy humans, but swelling is seldom seen in giraffes due to the stiffer and probably less permeable soft tissues. The swelling in human pathologies comes from the breakdown of one or more of these adaptations—in particular the frequent presence of a thick adipose layer that is both highly compliant and highly permeable. The higher compliance and permeability of subcutaneous adipose tissue might explain the strong correlation between the risk of developing lymphedema after axillary lymph node dissection and body-mass index at the time of surgery^109^.

It is important to note that chronic edema can lead to tissue stiffening as fibrosis occurs and the tissue interstitial matrix is modified. Likewise, there are age-related changes in the stiffness and thickness of the tissues that could have important implications for tissue mechanics. Stiffer, less permeable tissues would be less susceptible to large-scale pooling of fluid and would probably react more quickly to fluid transport when compression is applied. Unfortunately, last stage disease is often less treatable with compression because the fluid has been largely replaced with fibrotic adipose tissue that contains less fluid^14^. The conversion of the fluid phase of lymphedema to the fatty, fibrotic phase of advanced lymphedema is not well understood.

Late stage disease may also lead to wet, open wounds in which interstitial fluid may come into contact with the air. Under these circumstances, surface tension effects may become significant^110^. While beyond the scope of the current study, surface tension effects in an open wound may warrant further study.

As with any model, ours includes several assumptions and has notable limitations. First, the model does not explicitly include the blood vessels or lymphatic vessels. An interesting extension would consider how compressive stress in the tissue might alter venous valve function or impede blood or lymph flow. We also did not explicitly include the regulation of capillary pressure by precapillary sphincters. Failure of this mechanism could also lead to high capillary pressures and add to the source of interstitial fluid from the capillaries. This would produce increases in capillary pressure similar to those seen in venous insufficiency. In addition, it would likely be more realistic to use highly nonlinear mechanical properties for the tissues. The bulk modulus of the subcutaneous tissue has at least three distinct ranges: a stiff compressive range, a highly compliant range with pressure just above atmospheric, and a stiffening range for large levels of tissue dilation. In the current implementation such dramatic changes in properties led to numerical instabilities. Even with the linearized properties we employed, we were able to examine the behaviors of the system within each stiffness range that could then be compared between ranges. Finally, the assumed model of lymphatic pumping has the benefit of having only two simple parameters but does not capture the full range of behaviors that have been observed in vivo and ex vivo^111–113^.

The accumulation and removal of fluid from tissues is a complex process, and additional investigations can extend from our analysis. For example, the active effects of skeletal muscle contraction, especially during walking should dynamically change tissue mechanics and fluid pressures, and would be an interesting extension of the present study. Also, water in tissues can quickly change between mobile and immobile states as it is associated with or released by tissue hydrogels such as hyaluronic acid. This could also affect edema, and could be considered in future studies.

## Conclusions

We have shown that a simple set of length and time scales can clarify the relative importance of competing processes in fluid transport in the leg. The combination of high tissue permeability, high tissue compliance and low lymphatic clearance can lead to a positive feedback loop in which fluid can percolate so freely through the interstitial space that further buildup of fluid can occur in the lower leg due to gravitational pooling. Our model also shows that compression therapy can break this cycle by reducing large distance percolation of interstitial fluid.
